# Qualitative and Quantitative Analysis for Facial Complexion in Traditional Chinese Medicine

**DOI:** 10.1155/2014/207589

**Published:** 2014-05-22

**Authors:** Changbo Zhao, Guo-zheng Li, Fufeng Li, Zhi Wang, Chang Liu

**Affiliations:** ^1^Department of Control Science and Engineering, Tongji University, Shanghai 201804, China; ^2^Laboratory of Information Access and Synthesis of TCM Four Diagnosis, Shanghai University of Traditional Chinese Medicine, Shanghai 201203, China; ^3^School of Film & TV Arts and Technology, Shanghai University, Shanghai 200444, China

## Abstract

Facial diagnosis is an important and very intuitive diagnostic method in Traditional Chinese Medicine (TCM). However, due to its qualitative and experience-based subjective property, traditional facial diagnosis has a certain limitation in clinical medicine. The computerized inspection method provides classification models to recognize facial complexion (including color and gloss). However, the previous works only study the classification problems of facial complexion, which is considered as qualitative analysis in our perspective. For quantitative analysis expectation, the severity or degree of facial complexion has not been reported yet. This paper aims to make both qualitative and quantitative analysis for facial complexion. We propose a novel feature representation of facial complexion from the whole face of patients. The features are established with four chromaticity bases splitting up by luminance distribution on CIELAB color space. Chromaticity bases are constructed from facial dominant color using two-level clustering; the optimal luminance distribution is simply implemented with experimental comparisons. The features are proved to be more distinctive than the previous facial complexion feature representation. Complexion recognition proceeds by training an SVM classifier with the optimal model parameters. In addition, further improved features are more developed by the weighted fusion of five local regions. Extensive experimental results show that the proposed features achieve highest facial color recognition performance with a total accuracy of 86.89%. And, furthermore, the proposed recognition framework could analyze both color and gloss degrees of facial complexion by learning a ranking function.

## 1. Introduction

Nowadays, the Traditional Chinese Medicine (TCM) has become a global and important diagnostic approach in the medical field. In TCM, Inspection, auscultation and olfaction, interrogation, and palpation are four diagnostic methods to recognize the human pathological conditions. The traditional facial complexion diagnosis is an important part of inspection examination. The doctors can understand the physiological functions and pathological effects of human body through the inspection of complexion [1]. In retrospect, the inspection of complexion has been studied in TCM for a long time; however, it is still mainly based on the observations with practitioners' nude eyes. The disadvantage of nude observation is that it is likely to get inconsistent diagnostic results due to the large dependence on practitioners' subjective experiences and personal knowledge. Thus, it is worthwhile for TCM doctors and scholars to design an objective and reliable computer-assisted system for facial complexion diagnosis.

Recently, TCM experts stated that the notion facial complexion diagnosis includes two aspects: the facial color diagnosis and the facial gloss diagnosis. Therefore, by avoiding phrasing confusion, we declare here that we will employ the notion facial color instead of facial complexion, if it is basically studying one's facial color recognition. And we may still use the notion facial complexion, if it represents both color and gloss in this paper.

According to the TCM facial diagnosis theory, diagnostic significance of five colors implicates the correlation between five colors of facial skin and diseases. That is, the changes of facial colors can reveal pathological changes of different viscera and bowels with different natures. In addition, the lustre or gloss of skin refers to the reflective, shiny, and smooth characteristics of facial skin [2] which can help TCM experts to understand the states of visceral and bowels and the severity degrees of diseases.

To make it more detailed, the human facial color always has two cases: normal color and morbid color. The normal or healthy color is always further divided into two parts: normal individual color and varied normal color. The normal individual color refers to the normal natural color of skin that never changes in one's whole life due to ethnic and genetic factors. For the varied normal color, it is influenced by environmental factors and will change slightly with respect to the variations of the climates and seasons.

With these varied colors, which essentially are not morbid colors, the analysis of facial color will be difficult to put into practice; even the TCM practitioners find it hard to discriminate it from morbid colors. That will result in inconsistent diagnosis conclusion under different practitioners' observations. Just like the assessment about consistency of TCM experts' diagnosis results [1].

Besides, different from normal color, morbid color could be reflected on one's face on the condition that the pathological changes of five internal viscera (these are heart, liver, spleen, lungs, and kidney). The morbid color is classified to five colors: reddened facial color, bluish facial color, yellow facial color, pale facial color, and darkish facial color. Their correlation with syndrome in TCM can be simply stated as follows: reddened color implies heat syndrome; bluish color represents blood stasis syndrome; yellow color suggests dampness syndrome and deficiency syndrome, convulsive syndrome, pain syndrome, and cold syndrome; pale color indicates deficiency syndrome and cold syndrome; darkish color hints cold syndrome, blood stasis syndrome, fluid-retention syndrome, and the deficiency of kidney syndrome [3].

Furthermore, various lustre or gloss of skin also manifests different states of diseases. For example, if a patient is considered normal and with healthy facial color, the gloss of skin would be always bright and moist; that is, glossy skin indicates that patient is healthy [2]. However, for the morbid color patient, the gloss of skin reflects the severity of diseases. Patient whose facial gloss is bright and moist would indicate mild illness and easiness to cure no matter what color it is, while dull and dry gloss represent serious illness and difficulty to cure.

Generally speaking, it is significant to study the computerized facial color diagnosis more in-depth with multiple aspects. In the following, we will firstly review related facial color diagnosis techniques and systems in TCM. Then, the shortcomings of existing frameworks will be discussed, eventually leading to our motivation for building a facial color diagnosis with both qualitative and quantitative methods.

A large amount of pattern recognition and data mining technologies have been developed and designed for medical analysis and diagnostic standardization. The literatures [5, 6] propose novel algorithms for medical diagnosis and analysis. Recent machine learning algorithms, such as multi-label algorithm and multi-instance algorithm, have been introduced to deal with special medical data analysis [7–10]. Especially, some works about TCM four diagnostic methods [4, 11] have also been studied recently. There is an extensive literature on TCM four diagnostic methods, but we prefer to review just a few papers relevant with facial complexion analysis.

Early in China, some preliminary results have been reported in facial color analysis using colorimeter or infrared thermograph instrument. Unlike the previous foundation research, Liu and Guo [12] investigated the hepatitis diagnosis with face images by digital camera acquisition device. In their system, five facial regions are firstly segmented using skin detection, facial normalization, and horizontal position of the mouth, nostril, and eyebrow location. Then, the mean value of RGB color is taken as dominant color and *k*-nearest neighbour (KNN) for color classification. But the segmentation results are not always correct which needs to be adjusted manually. So Wang et al. [13] give an optimized version to improve the segmentation results and health versus hepatitis recognition accuracy. In addition, the fuzzy *c*-means (FCM) clustering method is introduced to extract the dominant color; finally six color spaces are considered and intensively analyzed for the best classification.

For the facial color analysis, the prior result [1] has established a five-color scale to measure facial color in RGB color space. Another research [14] firstly extracts 15 diagnostic feature points using AdaBoost and Active Shape Model (ASM), and, next, each rectangular region around each point will be used to calculate the facial color similarly as the above studies. More recently, Zhang et al. [2] analyze the facial color gamut and construct six centroids to calculate facial color distribution for health and several diseases classification. The texture features are firstly applied to their facial diagnosis system. For the facial gloss analysis, Zhang et al. [2] also present healthy classification using facial gloss information. Another main work for gloss classification is carried out using feature dimensionality reduction techniques [15]. However, some of the above works share several problems as follows.

- Adoptive dominant color as in [1, 13] is strongly depending on greater numbers of clusters which may be encountered failure to represent the morbid color in two cases: one case is the incorrect location of facial region that can derive biased dominant color; another is patients whose disease status is considered as mild, which will give rise to the facial morbid color slightly and inapparently. And, finally, it will extract inaccurate dominant color. One proposed way may overcome these problems as given in [14], but it is impractical to extract the basic skin color of upper arm for all the subjects.
- Only local and a few small facial regions used for the whole facial complexion diagnosis (including both color and gloss) may be difficult to reflect the general facial complexion; it also goes against the TCM overall facial observation theory. As mentioned in [1], the color of different facial regions on one's skin may be judged inconsistently even by the same TCM expert. But, using more or large facial regions might tend to approach general facial complexion and then would achieve better representation of one's overall facial complexion. In [14], 15 facial regions corresponding to TCM complexion-viscera are used as recognition samples, resulting in good facial color recognition accuracy. Although the facial region numbers may be large enough, the feature points' location algorithm still falls into complication.
- The most important issue is that all the above literatures are essentially qualitative analysis, which only aims to classify the facial complexions or diseases into their respective categories. But yet, in another sense, the severity of disease or the degree of complexion has not been studied as ever (e.g., one case is Yang jaundice with glossy skin and Yin jaundice with lustreless skin with respect to yellow color; for another case, flushed face and flushed cheek, which can be treated as different proportion of red color in the facial face, will indicate excess heat syndrome and deficiency heat syndrome, resp.).

To alleviate those discussed issues, we develop a novel framework for six facial colors diagnosis, which is built with four chromaticity bases and luminance distribution based on patients' whole face. Although it is mainly designed for facial color diagnosis, it also could be applied to the quantitative analysis for facial complexion involving color and gloss degrees.

On one hand, these developed four chromaticity bases are related to four facial colors (normal, redden, bluish, and yellow colors) and would be generated from the respective facial color gamut clustering. Those bases are still considered to be the dominant colors of facial skin but are achieved by means of two steps of fuzzy clustering. With this process, we can obtain more reliable dominant color compared with the works [1, 13].

Moreover, the luminance distribution would split up all chromaticity bases down into certain subbases for better representing the degree of luminosity gradient of our chromaticity bases. With the separated chromaticity subbases, the derived feature representation for the remaining two facial colors (pale and darkish colors) could be more distinctive to discriminate between the other four colors than the previous approach [2], which is basically constructed without considering the effect of luminance distribution on the facial color diagnosis.

On the other hand, not as some previous studies used to do, we would not expect to segment the patient' face image into specified facial regions but to extract one's whole skin color as holistic representation. That is to say, the region segmentation procedure as done by the previous studies [1, 2, 12–14] could be bypassed in our framework. Nevertheless, for the improvement of classification performance, we also find that the combination of locally weighted region and global representation could achieve more significant improvement than only the local or global approaches.

In this regard, facial color distribution would be reliably estimated using our well-established holistic facial complexion representation. This might be more in accordance with TCM overall concept and diagnosis in practice. Furthermore, it would be possible to analyze both qualitative and quantitative issues through our proposed framework.

The remainder of the paper is organized as follows.  Section 2 describes the novel facial complexion feature representation and how it is applied to quantitative analysis for both color and gloss degrees. Extensive experimental results and several improvements of the color classification are presented in Section 3. Finally, Section 4 draws some conclusions and future directions.

## 2. Methods

**Figure 1:**
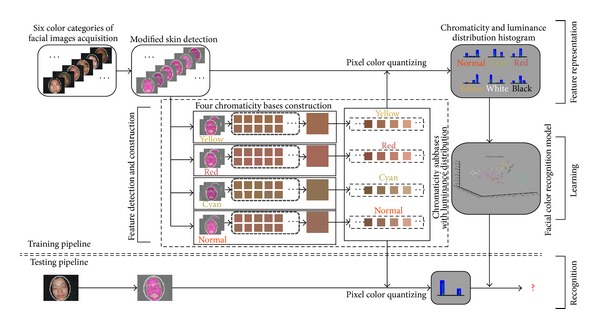
Pipeline of the proposed facial color classification framework.

This section gives detailed descriptions of our developed facial complexion diagnosis chain, which is briefly summarized in Figure 1. As the pipeline shows, our framework is composed basically of four main stages as follows.

1. At the beginning, four chromaticity bases and their luminance distribution will be constructed on their corresponding facial color images, that is, feature detection and construction stage illustrated in the pipeline.
2. Then, the designed feature will quantize facial skin color to form complexion distribution on all collected facial images, called feature representation stage.
3. At the learning stage, a Gaussian Kernel (or RBF kernel) Support Vector Machine (SVM) with the optimal parameters is employed to build the facial color model.
4. In the final recognition stage, for any testing facial image, the learnt model will determine its facial color category.

In addition, beyond facial color recognition, we further make quantitative analysis of color and gloss degrees for each patient using our established feature representation. Subsequently, we present all procedures step by step.

### 2.1. Acquisition System and Data Description

The facial image acquisition system is the same as our previous one reported in [11]. This acquisition device mainly consists of annular LEDs and digital camera which are chosen with lots of trials and errors. More specially, because a digital image is produced highly based on the light source and camera, the achieved image might be color distorted, if no color correction was taken under nonstandard light source. In order to reduce the effects of light source, both illumination characteristics of light source and imaging characteristics of camera have been designed experimentally to obtain acceptable facial image. The illumination characteristics have been assessed and analyzed by studying various light sources, and, finally, the best light source with appropriate illumination characteristic (color temperature value is about 5600 K, Ra = 90) is determined as our light source. As for the camera, the imaging characteristics of several available cameras at different modes were also compared and evaluated. Then the best camera (Canon PowerShot series S3 IS) on the best mode with white balance is set up. More details about our acquisition system could be found in [11].

After the development of facial acquisition system, different patients' facial images are obtained and diagnosed on the color corrected monitor by three senior TCM physicians, totally containing six color cases. Such facial images would be recorded with its corresponding color category if more than 2 physicians would make the same diagnosis result.

Consequently, our facial color dataset is built up with a total of 122 cases, containing 24 normal color images, 15 for bluish, 18 for reddened, 24 for yellow, 21 for pale, and 20 for darkish color images. This dataset seems a little small compared with the previous works. This is mainly because our collected facial images need to be filtered by TCM experts firstly if the images are unavailable and then diagnosed by senior TCM physicians. Thus only a small number of facial images are obtained, if the preliminary images are not enough. And, for further research, it is better to collect more data in the future. For color recognition, normal color would be tagged with normal, and all other morbid facial colors (bluish, reddened, yellow, pale, and darkish) would be tagged with its similar color spec named cyan, red, yellow, white, and black. These tags or labels are also illustrated in the framework of Figure 1. In the subsequent parts, for convenience, we would prefer to describe those six facial colors in TCM using their label names.

### 2.2. Skin Detection and Fine-Tuning

Different from the previous studies, our basic idea is to study the overall complexion condition of patients' face, which may avoid the influence of inaccurate or insufficient local region location and, instead, strengthen the expression of facial complexion in global situation. To meet this goal, an automatic and efficient skin detection approach which is published recently in [16] will be introduced. To put it simply, this approach is a fusion strategy that firstly utilizes a smoothed 2D histogram with I and By channel in log opponent chromaticity (LO) space, and then combines Gaussian Mixture Model (GMM) for modeling the threshold of skin-color distribution. Although there exist a large amount of skin-color detection solutions, we still adopt this fusion version due to two benefits: (1) it is able to cope with the variety of human skin colors even across different ethnic origin, which is especially fit to detect facial skin with different color variations in TCM; (2) it can be managed with low computational cost as no training stage is required.

Nonetheless, one case has been long-term existed in skin detection, which is known as the prone false skin detection of lip. This is presumably because the facial color gamut is highly overlapped with lip color gamut, leading to unexpected skin detection result, especially when skin detection approach is performed only with color feature. Such case is not expected to occur in our facial color recognition system because of its interference on original facial color distribution. Thus, we strongly hope to do a fine-tuning processing so as to crop the corrected skin and discard the mouth region. But accurate mouth segmentation algorithms may be bound up with high computational cost; it is desirable to consider the algorithm characterized by low computational cost and coarse segmentation, such as returning a simple rectangular or circular window on lip. In other words, we prefer to detect mouth region rather than segment the lip along the boundary.

Fortunately, the appearance of facial profile and components (eyes, mouth, and nose) is distinctive from each other, arousing vast works on facial detection. Of these, one face detection approach, known as Viola and Jones' object detection framework [17], shows its excellent performance with low false negative rate and rapid face detection. The detector is firstly done by fast extracting a number of overcomplete haar-like features using integral image, across different scales and different spatial positions on image. Then AdaBoost learning algorithm will be used to select a low number of critical visual features from above large set of features. Finally, a cascade scheme for combining classifiers is built to quickly discard background regions of the image. Another extended work [18] introduces a novel set of rotated haar-like features, which significantly enriches original features and further improves the overall performance. Hence, in this paper, we expect to introduce this well-known framework for localizing facial mouth.

Now, the main issue is how to build a facial mouth detector using Viola and Jones' object detection framework. To be honest, it is time-consuming to prepare the training data and then train a cascade classifier for specific application. Thankfully, the OpenCV community shares a collection of public domain classifiers for facial processing which contains the facial mouth detector. All those trained classifiers are available in the haar cascades repository [19].

**Figure 2:**
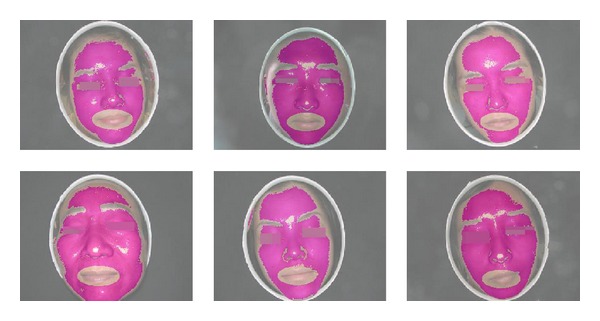
Six typical facial skin regions after skin detection and fine-tuning.

Since our facial images are all in frontal view, it is compatible to carry out the detection directly based on this public mouth detector. Then, from the mouth detection result, it should be noted that the discarded mouth region might also remove some skin colors around the lip. Nevertheless, it would not impact the estimation of overall facial color distribution due to such minority skin pixels. This is one reason why we prefer to perform mouth detection instead of accurate but time-consuming lip segmentation. Another reason to employ the Viola and Jones' detector is that it is extremely rapid and reliable, which has been extensively used in computer vision research. Some examples of skin detection and its fine-tuning results are illustrated in Figure 2.

After the skin fine-tuning stage, skin color denoising process would be done to remove the scattered spots (e.g., black moles) on the skin and a small minority of hairs covering the forehead, which is characterized by higher or lower luminance value. We simply employ the cumulative histogram to calculate the gray distribution and remove the top and bottom gray level satisfying certain proportions.

### 2.3. Chromaticity Bases with Luminance Distribution Construction

Once we got the skin region, we can turn to construct the chromaticity bases and luminance distribution. This is called the feature detection and construction stage. In this stage, three steps of the feature construction are performed: color space transformation, chromaticity bases construction, and final feature representation with luminance distribution.

*Color Space Transformation.* In order to realize the representation of chromaticity and luminance separately, the transformation from RGB color space to CIELAB color space is realized. CIELAB is a color-opponent space with *L* component for luminance and remaining components (*a* and *b*) representing the chromaticity. Since the CIELAB color space is made on the basis of CIEXYZ color space, we need firstly to transform the RGB color space to CIEXYZ color space [20]. The transformation formula is given as below follows:

(1) $$\begin{array}{c} \left[\begin{array}{c} X\\ Y\\ Z \end{array}\right]=\left[\begin{array}{ccc} 0.4124 & 0.3576 & 0.1805\\ 0.2126 & 0.7152 & 0.0722\\ 0.0193 & 0.1192 & 0.9505 \end{array}\right]\left[\begin{array}{c} R\\ G\\ B \end{array}\right]. \end{array}$$

Then, the transformation from CIEXYZ color space to CIELAB color space is done by the following formulas:

(2) $$\begin{array}{c} L^{⋆}=116f\left(\frac{Y}{Y_{n}}\right)-16\\ a^{⋆}=500\left[f\left(\frac{X}{X_{n}}\right)-f\left(\frac{Y}{Y_{n}}\right)\right]\\ b^{⋆}=200\left[f\left(\frac{Y}{Y_{n}}\right)-f\left(\frac{Z}{Z_{n}}\right)\right], \end{array}$$

where

(3) $$\begin{array}{c} f\left(t\right)=\{\begin{array}{cc} t^{1/3} & \text{if}\;t>{\left(\frac{6}{29}\right)}^{3}\\ \frac{1}{3}{\left(\frac{29}{6}\right)}^{2}t+\frac{4}{29} & \text{otherwise}. \end{array} \end{array}$$

Here, *X*_*n*_, *Y*_*n*_, and *Z*_*n*_ are the CIEXYZ tristimulus values of the reference white point. Often, its values are assumed as *X* = 95.047, *Y* = 100, and *Z* = 108,883 relative to CIE standard illuminant D65. Based on the color space transformation, all of skin pixels would be converted and applied to construct our feature representation. 

*Chromaticity Bases Construction.* In this step, four chromaticity bases are constructed using two-level clustering on the CIELAB color space. The objective of establishing these four bases is to explore the basic chromaticity with respect to each facial color. Based upon basic chromaticity, it would be possible for us to assign the skin pixels to its closely measured distance. And, thus, final established feature representation could be regarded as the facial chromaticity ratio, which would be easy to distinguish among different facial colors. (e.g., cyan facial color would have a large number of skin pixels approaching cyan chromaticity but less number of pixels getting close to other chromaticity bases.)

This idea is mainly inspired from the bag-of-words method in computer vision [21]. And similar approach in facial diagnosis has been introduced by Zhang et al. [2]. Our constructed bases are similar but exactly not identical. In their approach, six centroids (similar as bases) are manually selected from the facial color gamut. But in our framework, we tend to automatically select four bases by clustering algorithm and expect to make full use of label information of given facial color category.

To be more specific, from the previous studies [1, 13], the dominant color of facial skin is treated as the greater number of cluster center by FCM. In other words, they assumed that the potential facial color in TCM appeared in the dominant color, but, exactly in practice, dominant color may fall into the minor number of cluster center as we stated before. Besides, TCM theory considers the facial color consisting of two kinds of colors: facial color and basic skin color [14]. In general, we expect to assume that facial chromaticity basis basically appeared in dominant color or subdominant color.

According to this assumption, the purpose of our chromaticity basis extraction framework is to find out facial color chromaticity regardless of it may lie in facial dominant or subdominant chromaticity. So, the major construction procedures of chromaticity bases can be summarized as two levels of clustering:

- *pixel-level clustering*: for each patient's facial image, the dominant and subdominant chromaticities of facial skin are extracted by FCM clustering; that is, cluster center number is set to 2; this step is the same as the one in the previous studies, but we further drive it to generate the color chromaticity base with second level clustering;
- *chromaticity-level clustering*: based on dominant and subdominant chromaticity, the second clustering is performed; then, in this step, chromaticity base chooses the cluster center with a greater number of members by FCM; it should be noted that all bases are built under respective facial color categories. Thus, we will obtain four bases for four facial colors (normal, red, cyan, and yellow colors).

As seen in Figure 1, inside the feature detection and construction stage titled “four chromaticity bases construction,” we demonstrate the procedures to generate four bases. It can be illustrated that all bases are constructed separately, so each basis will not be affected by other colors. In the first level clustering, it would be possible to collect multiple potential facial color chromaticities within dominant and subdominant chromaticities. This process makes sense because images with the same color class can extract similar color chromaticity in dominant or subdominant chromaticity, no matter which one is our expected chromaticity. Then, it would be reliable to choose the final cluster center with a greater number of members as our final color chromaticity basis in the second level clustering.

**Figure 3:**
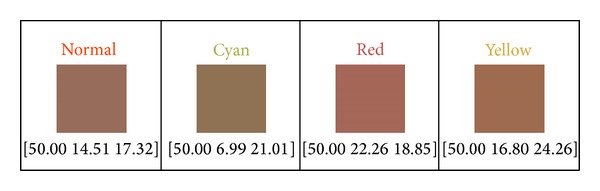
Four chromaticity bases constructed by the proposed approach.

We also give those produced bases in Figure 3 their respective chromaticity values at the bottom. Values inside the square brackets represent* [L*, *a, and b]* (the component values in CIELAB color space). But the *L* component is meaningless here, just for color display only. Because only two chromaticity values in CIELAB color space are unable to produce color, so we simply set the luminance value as 50 on all chromaticity bases for pure illustration. 

*Luminance Distribution Construction.* Using the above four chromaticity bases, four facial colors can be discriminated from each other. However, the remaining two facial colors, white and black, would fail to be recognized from others. This is reasonable mainly because their distribution of chromaticity gamut is highly overlapped with other four facial colors, leading to impossibly distinguished characteristic through the chromaticity only. Fortunately, the luminance characteristics of them are diametrically opposite, which is easy to represent by the *L* component of CIELAB color space. This is our basic motivation of building white and black colors feature representation.

In support of this goal, the luminance distribution should be constructed to integrate the bases we designed before. Recall that four chromaticity bases are constructed only by the *a* and *b* components of CIELAB color space, one conceivable way for constructing luminance distribution is by splitting up the basis into several subbases by spanning a range of luminance. By this method, final feature representation could be characterized by two advantages:

- for each chromaticity base, it can represent the chromaticity distribution of facial skin color, making four facial colors separable. And, moreover, we could make quantitative analysis for facial color degree;
- for each subbase, the luminance distribution would be calculated for each chromaticity base, providing a solution to easily recognize white and black colors. It would also support us to make quantitative analysis for facial gloss degree.

The designed chromaticity subbases with luminance distribution have been shown in Figure 1. We simply set the range of luminance by default (*L* component value ranges from 0 to 100) and set the interval value of adjacent luminance as ten. Therefore, totally a vector length of 40 features is obtained for each facial color image. Although this process is coarse at first, we will refine it to explore the best luminance distribution by experimental comparison.

### 2.4. Facial Complexion Representation

By the well-established subbases with both chromaticity distribution and luminance distribution, the facial skin pixel color assignment is performed on each image, thereby quantifying the facial complexion and building our feature representation. We still call the feature representation as facial complexion feature instead of facial color feature due to its expression ability for both color and gloss, so enabling us to make quantitative analysis for the facial complexion degree.

For every patient's image, its facial skin pixels are assigned to nearest subbases using color distance measurement in CIELAB color space. After the assignment, a complexion histogram is formed and would be used to measure for both chromaticity and luminance distribution. In order to represent the histogram as the facial complexion ratio distribution, and especially for quantitative complexion analysis, histogram normalization is carried out to achieve this purpose.

**Figure 4:**
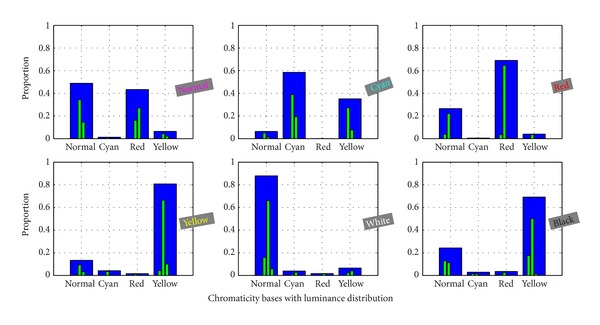
Six typical facial complexion histograms.

Figure 4 shows six facial color categories of established complexion histograms (which has been adjusted with the optimal luminance distribution). Four chromaticity bases are rendered as blue color, and subbases for luminance distribution are rendered as green color. In the complexion histogram, the highest blue peaks of chromaticity bases correspond to dominant color of facial skin, and subdominant color would be identified by the second highest blue peaks. For these subbases rendered with green color, it denotes the luminance distribution with respect to each chromaticity base. Such process would be more efficient to represent the facial complexion than without consideration of luminance distribution. Furthermore, seen from the histogram, white facial color would hold higher values in luminance distribution at each chromaticity base, while black facial color are always with lower luminance values. Essentially, the proposed feature is only involving color feature. We note that the texture and the shape features are widely used in image representation. But considering the facial color recognition, the texture and the shape features seem not as important as the color feature in the description of facial colors. Thus, in this framework, we put the texture and the shape features aside.

Benefiting from the proposed feature representation, colors of normal, cyan, red, and yellow which are separable on chromaticity could be easier discriminated from each other. Besides, white and black colors which are easily classified by luminance values could be correctly recognized. More important advantage from the combined chromaticity and luminance distribution is probably to analyze color and gloss degrees in quantitative way, given in later stages.

### 2.5. Facial Color Recognition

Before we discuss the facial color recognition model, it is noted that the proposed feature is unnecessary to be normalized along each feature dimension before modeling. This is not as we usually do when building a machine learning model. Because the constructed features are normalized histograms, denoting the color proportions on one's facial skin. All feature values have normalized from 0 to 1. Especially, we would not perform normalization process again, avoiding destructing the color proportions information.

In fact, facial color recognition is basically considered as a multiclass classification problem. There are various classifiers available for tackling this problem. So we have compared SVM with several type classifiers (e.g., *K*-nearest Neighbor and Nave Bayes); the classification result shows that the SVM could achieve better performance than compared classifiers. This is the main reason why we prefer to adopt SVM for facial color recognition here.

It is easier to implement the SVM model with LIBSVM toolbox [22]. For multiclass problem, LIBSVM supports one-against-one strategy for multiclass SVM with building several binary classification models. In our paper, 6 facial color labels would produce 15 pairwise SVM models. And, finally, any test facial color will be assigned the maximum voting label, where each SVM model votes for one label. In addition, we choose a Gaussian kernel SVM with the optimal penalty factor *C* and width parameter *γ* in our experiment.

### 2.6. Facial Complexion Quantitative Analysis

The facial color recognition model is constructed for qualitative analysis of facial complexion, especially for facial color. But for patients belonging to particular facial color category, the severity or degree of disease is unknown for the previous studies. In addition, given a set of patients with specific facial color category, the relative degrees of facial color for patient have not been studied ever. These relative degrees mean that the patient's facial color or gloss degree is adjacent to someone. So in this paper, after facial color recognition stage, we also explore some experiments to make quantitative analysis based on our feature representation. Besides, this quantitative diagnosis has not been developed yet, we attempt to implement some unsupervised methods to do some preliminary trials.

To make quantitative analysis, we assume that facial color pixel numbers of specific color could represent the degree of color, and glossy skin is characterized by reflective, shiny pixels. Thus, after performing facial color recognition, two situations could be studied for quantitative analysis: color degree without luminance distribution and gloss degree without chromaticity distribution.

However, we should notice that our feature representation for facial complexion can be regarded as chromaticity distribution for four facial colors (normal, cyan, red, and yellow). For each chromaticity, it is splitting up by luminance distribution, making it possible to recognize black and white facial colors. Due to the characteristic of our feature, both white and black facial colors are neglected to conduct quantitative analysis for color degree. On the contrary, gloss degree will take all facial colors into consideration.

#### 2.6.1. Color Degree of Quantitative Analysis

Color degree presented here is defined as the quantity of color pixels in one's facial skin. This definition makes sense because large quantities of color indicate obvious color reflection, and corresponding to the degree of pathological changes of five internal viscera.

Therefore, our method for color degree quantitative analysis is to build the scope of pixels numbers on the specific color. In other words, the ranking function of color degree is learnt. So for any patient, its color degree would be estimated by this ranking function.

To achieve each color ranking function, we firstly quantify facial skin pixels into a chromaticity distribution histogram without splitting up by luminance distribution, deriving a vector length of four features. The histogram feature indicates the proportions of each color on facial skin. Then given a set of the same facial color category of patients, their corresponding facial color proportion values in histograms are used to determine facial color degree ranking function. In this paper, we simply utilize normalization to quantify color degree into range from 0 to 1. Finally, for any patient whose predicted facial color belongs to given color, their facial color degree would be estimated by built ranking function. Besides, its relative color degree patients are also obtained with this ranking strategy.

#### 2.6.2. Gloss Degree of Quantitative Analysis

Gloss degree is defined as the pixel quantity of higher luminance value; higher degree suggests glossy skin. This definition is raised by the characteristics of glossy skin mentioned before. With gloss degree, TCM experts could analyze the physical condition of patient. The facial skin of normal color patients would always be glossy; so it also provides complementary information to recognize normal facial color. For morbid facial color, higher gloss degree indicates mild illness and is easier to cure.

Similar to facial color degree, gloss degree is also quantified by a ranking function with normalization. But the gloss representation is slightly different due to its definition. Given all facial complexion histograms we used in recognition stage, the four bases describing chromaticity distribution will be merged. We sum over these bases and obtain a novel histogram only with luminance distribution. This is opposite to color degree which only describes chromaticity distribution.

To achieve gloss degree estimation, we should firstly compute gloss score for derived histogram feature. This is done by summing over all luminance values with weight values in luminance distribution. Those weights are selected by principle that higher luminance values are assigned to large weights. After computing gloss scores for all given patients, the gloss degree ranking function is built by normalized all gloss scores. Then, the gloss degree or relative gloss degrees could be estimated by ranking function for any patient.

## 3. Experiments and Discussions

The experimental images are collected by Shanghai University of Traditional Chinese Medicine. All images are diagnosed by three practitioners and labeled only if at least two of them made consistent conclusion. Eventually, we have established a small scale facial color data set, including 122 subjects with two facial color cases. One is normal or healthy facial color (24), another is morbid facial color with 5 categories: cyan (15), red (18), yellow (24), white (21), and black (20), respectively.

### 3.1. Experimental Setup

In our experiment, leave-one-out cross validation (CV) is used for evaluating the model performance. To build the facial color recognition model, all the training images will be employed to construct the facial chromaticity bases and calculate complexion histograms for all images in the data set. Our images are collected by high definition camera (5-megapixel), which requires high computational cost to manage these facial images. So the height and width size of each facial image are resized by one-eighth of origin, deriving one-sixtieth reduced size of every facial image.

For the SVM with RBF kernel classifier, we use grid search to obtain the optimized parameters and select *C* and *γ* values from the range 2^−8^ to 2^15^ and 2^−8^ to 2^8^, respectively. Then, in each fold of leave-one-out CV, we yield the best classification performance with these combined optimal parameters. Finally, the evaluation criteria are calculated based on the test results.

### 3.2. Evaluation Metrics

There are many criteria used for evaluating model performance, we consider several generalization measurements which is more reliable to assess multiclass problems [23].

Given the confusion matrix *A* and classes *C*, we can derive one-versus-all confusion matrix for each class; then the generalized precision and recall criteria formulas for each class are defined as follows:

(4) $$\begin{array}{c} {\text{Precision}}_{i}=\frac{\text{T}{\text{P}}_{i}}{\text{T}{\text{P}}_{i}+\text{F}{\text{P}}_{i}},\;\;{\text{Recall}}_{i}=\frac{\text{T}{\text{P}}_{i}}{\text{T}{\text{P}}_{i}+\text{F}{\text{N}}_{i}}. \end{array}$$

Here, TP_*i*_ are the number of true positives for *C*_*i*_ at confusion matrix element *A*_*ii*_, TN_*i*_ are true negative numbers, FP_*i*_ indicate false positive counts, and FN_*i*_ indicate false negative counts, respectively.

Then, the overall *F*-measure score can be calculated with precision and recall, which could measure the overall classification quality of multiclass problems. Especially, it contains two types of *F*-measure, called microaverage and macroaverage. In addition, we also present confusion matrices visualization both for recall and precision. 

*Microaverage and Macroaverage F* *-Measure Scores.* The *F*-measure score is essentially a weighted combination of precision and recall, which is defined as

(5) $$\begin{array}{c} F\;\;{\text{score}}_{M}=\frac{\left(\beta^{2}+1\right){\text{Precision}}_{M}{\text{Recall}}_{M}}{\beta^{2}{\text{Precision}}_{M}+{\text{Recall}}_{M}}, \end{array}$$

where *β* is generally considered as 1. If the indice *M* indicates microaverage, the formula would derive the microaverage *F*-measure score; then its precision and recall are then computed as

(6) Precisionmicro=∑i=1LTPi∑i=1L(TPi+FPi)Recallmicro=∑i=1LTPi∑i=1L(TPi+FNi).

In microaverage, it can be seen that precision and recall are calculated over all samples. So it gives equal weight to every sample, which is useful to measure the performance on the common numbers of classes.

If the indice *M* indicates macroaverage, we obtain the macroaverage *F*-measure score whose precision and recall are computed in another way as below:

(7) Precisionmacro=1L∑i=1LTPiTPi+FPiRecallmacro=1L∑i=1LTPiTPi+FNi.

From the above formulas, we can figure out that the macroaverage is the harmonic average across each class, which would give equal weight to every class regardless of the class numbers. In this way, the macroaverage *F*-measure score could evaluate the performance on rare numbers of classes. So the microaverage and macroaverage are complementary to each other, and both of them are informative for performance evaluation. 

*Confusion Matrix.* Besides, we introduce two confusion matrices for recall and precision. The reasons employ two metrics of confusion matrix for facial color recognition model are as follows:

- the confusion matrix for recall could clearly show the proportion distribution of predicted facial colors with respect to each actual facial color, which illustrates the model performance for each actual facial color;
- while the confusion matrix for precision could demonstrate the proportion distribution of actual facial colors with respect to each predicted facial color, which measures the quality of each predicted facial color.

In the following, we perform extensive experiments to study the performance of our proposed facial color framework. Besides, not only will we present both qualitative and quantitative experimental results, but also some more details about the improvement of our framework are explored, which yield excellent performance compared with the previous methods.

### 3.3. Preliminary Results on Qualitative Analysis

In this section, some preliminary results in qualitative aspect are produced and compared using above metrics. It should be noted that qualitative analysis mentioned here is referred to as facial color classification problems. The quantitative analysis is considered as the severity or degree of one's facial complexion, which will be presented later. 

**Figure 5:**
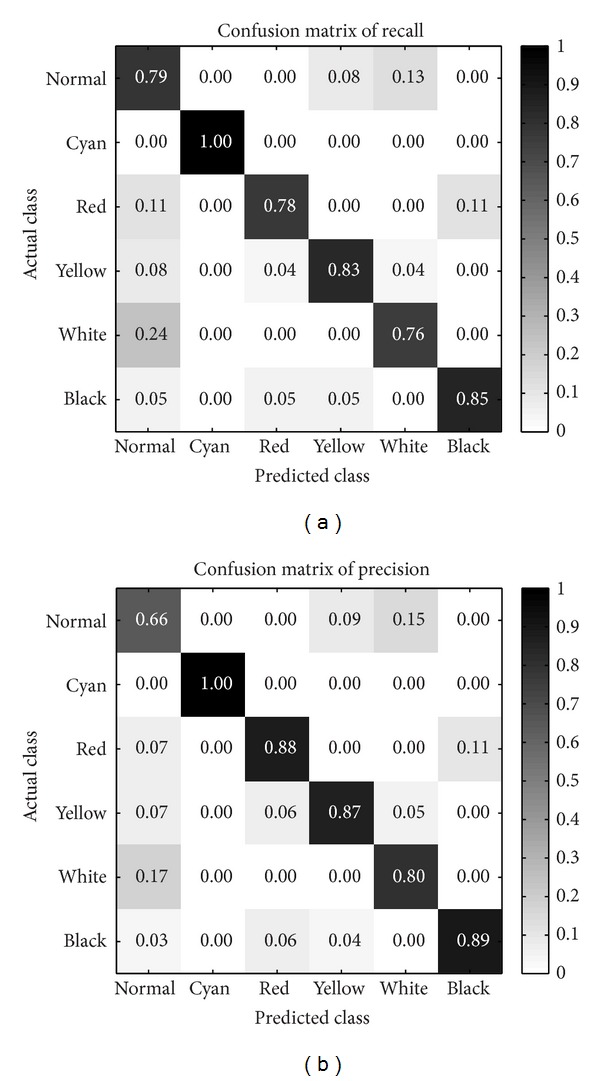
The confusion matrices produced by our model.

*Each Facial Color Category Classification Performance.* After performing leave-one-out CV, confusion matrices for recall and precision are firstly calculated, as shown in Figure 5. The row of confusion matrix means the actual class in database, and the column means the predicted class by our model. Each table cell indicates the ratio value according to specific metrics, and, the darker the table cell, the higher the ratio. Left confusion matrix is computed for recall metrics and right one for precision metrics. It should be noted that the confusion matrix for recall will only make sense along the column, while precision makes sense along the confusion matrix table row.

According to the normal and white rows on confusion matrix for recall, we observe that normal facial color is slightly prone to be predicted as white and vice versa. Red facial color images have some cases misclassified as normal or black facial color. And yellow and black facial colors achieve better recall values than normal and red.

However, all cyan facial color images have been recognized perfectly. This result for cyan is presumably because its chromaticity base is highly distinctive to other colors which might be easier to be discriminated. But another potential reason may give rise to this outstanding result only for cyan facial color is as follows: cyan color image numbers of our database are not enough (only fifteen images). So the classification performance of cyan facial color on a larger amount of images is still unknown for us.

Observing the right confusion matrix for precision along the row, we note that the worst quality of predicted classes is shown on normal facial color. Predicted normal facial colors are almost misclassified across all other colors, especially those inclining to white facial color. But our model achieves superior precision for other predicted colors.

From Figure 5, we discover that normal facial color is the most difficult class to be recognized. Indeed, in the TCM theory, normal facial color is defined as hybrid colors composed of red and yellow chromaticities with higher luminance. So it is reasonable to be misclassified as white, red, or yellow for normal color, as shown in our confusion matrix results. This also makes us consider how to build the normal color chromaticity base, but we have not studied it in this paper, we hope to put it further in our future study.

From another perspective of each facial color classification performance, it is also considerable to prove the argument we highlighted before that our bases is reliable to extract the dominant color from facial skin, with two-level clustering method. But the chromaticity bases are constructed only with four facial colors. To this end, we ignore white and black facial colors and use only four chromaticity bases histogram to performance classification. For classification, the classifier SVM is not adopted; four facial colors will be predicted by the dominant color or peak bin in histogram.

**Figure 6:**
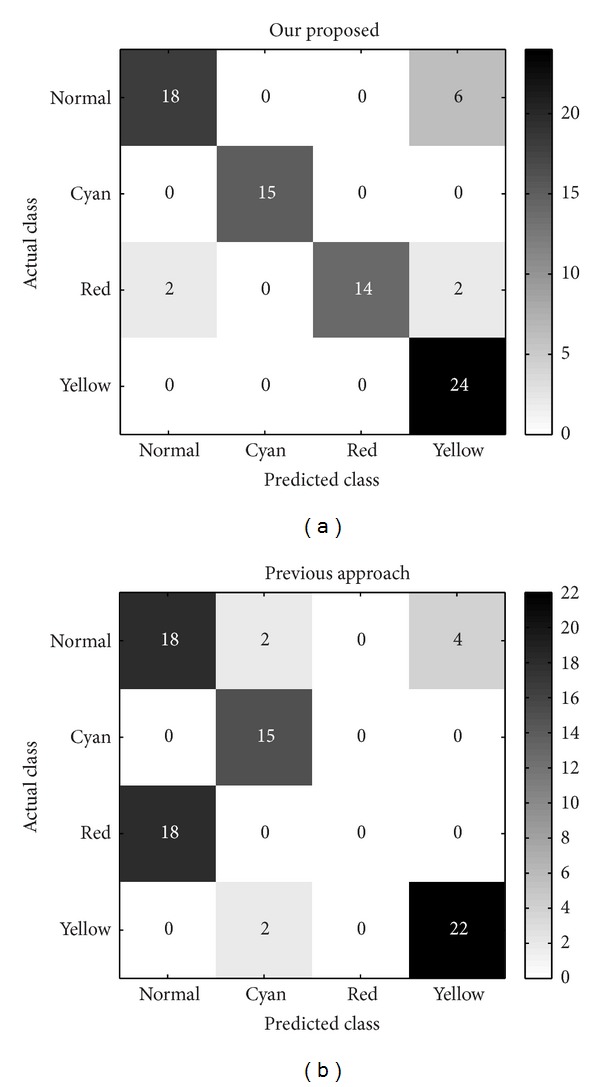
Comparisons of dominant color extraction methods on four facial colors.

For comparison, another chromaticity bases are constructed without two-level clustering, just clustered by FCM once as in [1, 13] and then calculates mean value of large cluster as dominant color. We show confusion matrix counts comparison between our proposed and the previous methods in Figure 6, each cell record the predicted facial color counts with respect to actual class. Compared with these two tables, the classes normal, cyan, and yellow are quite competitive between two methods. But the previous approach incorrectly predicts all red facial colors as normal class. This happens presumably because red facial color images in our database are hard to distinguish from normal facial color. And then the previous method may probably derive similar chromaticity base between normal and red facial colors. But our proposed dominant color extraction for constructing chromaticity base could avoid this situation with two levels clustering, due to the full use of label information of each class. Moreover, with such simple classification criteria and lower dimensional features, our total accuracy for four facial colors recognition reaches 87.65%, but the previous method only achieves 67.90%.

*Overall Facial Colors Classification Performance.* We then present the overall facial color classification performance of our proposed complexion feature representation. For the purpose of validating the distinctive feature representation, we proposed several previous features [1, 12, 13] for facial color recognition that are implemented in this paper. All features are extracted on whole facial skin pixels. In this process for compared features, we expect to only explore the facial color representation ability regardless of the influence of other factors, such as local regions versus whole face.

On the implementation of [1], six facial color RGB values are derived and used to measure the similarity of each test facial color, which could be similarly treated as the color distribution feature. But, slightly different from their classification strategy, we implement the distance metrics using nearest neighbor instead of radius scope, due to its higher performance with our database. In [13], dominant color of facial skin pixels is extracted in RGB color space and transformed into other five color spaces; finally, 18 features are obtained for whole face. And, for [12], average RGB values of whole facial pixels are used as color feature. Sincerely, Wu et al. [14] may achieve superior accuracy for facial color recognition problem, but the operation to collect skin color of patients' upper arm is impractical for our current database, so we do not implement it for comparison.

We list our comparison results in Table 1, where “*F*_micro_,” “*F*_macro_,” “Pre_macro_,” and “Rec_macro_” represent microaverage *F*-measure score, macroaverage for *F*-measure score, precision, and recall. We do not present microaverage precision and recall because they are numerically equal to microaverage *F*-measure score according to their definitions. From classification evaluation, we find that our complexion representation could achieve highest performance. Even though our existing facial complexion representation could perform better than the previous methods, we still consider some possible improvements for our framework. So in the following section, we discuss some adjustable parameters and regional fusion strategies to further improve our facial color diagnosis framework.

### 3.4. Improvements on Facial Color Classification

Above results are preliminary since some factors are still without consideration, so we would like to explore more details about our facial complexion representation, which could not only reduce the dimension feature representation but also improve our model recognition rate. 

*The Effect of Luminance Distribution.* Some previous qualitative results are obtained by setting the interval of adjacent luminance as ten by default. Since the luminance value of *L* component in CIELAB color space ranges from 0 to 100, so a vector length of 40 features is formed for each facial complexion feature representation. But it is considerable to study how many intervals to quantify one's facial luminance would provide higher discrimination; so the finer quantization of the luminance distribution is selected by experiment comparison.

From the preliminary parameters setting of luminance distribution spacing, we have found that extreme luminance value of facial complexion histogram is nonexistent. It happens because we have conducted the preprocessing step to remove spots (e.g., black moles) on facial skin. Thus, the refined luminance range is selected from value 25 to 95 based on the observation of final histograms.

**Figure 7:**
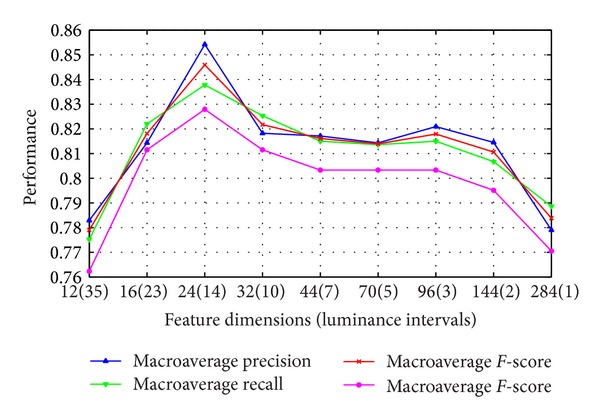
Classification accuracy with different luminance distribution intervals.

Then the interval of adjacent luminance is determined experimentally. In Figure 7, we show the classification accuracy results over luminance intervals from 1 to 35, deriving feature dimensions from 12 to 284. The experimental intervals are selected if the luminance range can be divisible. In addition, interval values 23 and 3 are also performed due to some large spacing at luminance intervals or feature dimensions. This selection of feature dimensions is not uniform but rational because the main effect of luminance distribution for facial color classification is the luminance interval number. For easy calculation and even intervals, we take the given intervals as our testing experiment. In summary, the best classification performance is achieved when luminance interval is set as 14. So totally, only 24 feature dimensions and luminance ranging from 25 to 95 are formed to represent facial complexion and build recognition model. We also find that extreme luminance intervals would achieve inferior classification performance in our experiment. 

*Holistic Representation with Weighted Local Spatiality.* Sincerely, studying on the local regions is reasonable for facial color. According to TCM experts, color recognition could behave well just on some local regions (e.g., cheek regions). To explore the facial color expression ability of different local regions, we start to analyze facial color recognition performance on different local regions.

**Figure 8:**
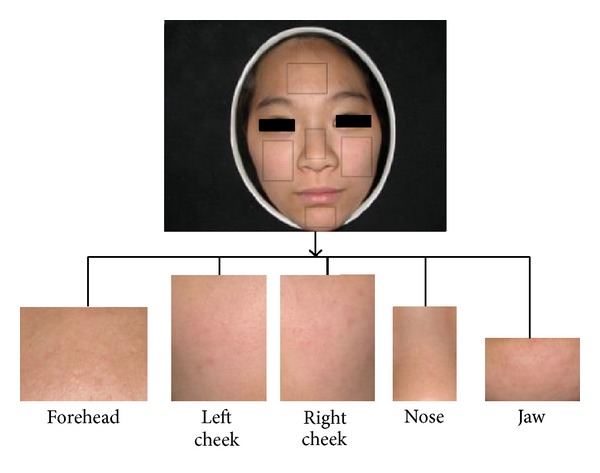
An example of facial image segmentation.

For making experiments on this case, we simply do the local regions segmentation manually, as shown in Figure 8. We partition the whole face into five regions similarly as the previous works [1, 2, 12, 13]. Then each region is used to extract specific features based on aforementioned experiment (total 24 features used) and perform classification separately. Finally, we list the classification results in Table 2. From the classification performance on different local regions, we find that cheek regions could achieve more superior results than other regions. This result is consistent with TCM experts' experiences that cheek regions provide more facial color information than others. In addition, we also list our whole face result here. It achieves highest performance than any local region strategies for classification. This also proves our argument that whole facial color representation would be better than only local regions.

Therefore, different regions contribute different facial color information, it makes us think that spatial distribution information might be useful to represent facial color. Considering our previous feature representation, two levels of information have been described on whole face: chromaticity-level and luminance-level distribution for the histogram. But our features for facial color have not yet contained information about regional level (or spatial level) on different local regions. To this purpose, the regional level color distribution is built to achieve a novel holistic representation. So we need additional stage to segment local regions and rebuild our facial complexion representation.

**Figure 9:**
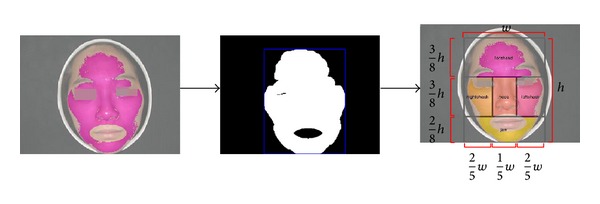
The flowchart of local regions segmentation.

Our facial images are all frontally scanned with small pitch; so for avoiding complicated local region segmentation process, a simple partition procedure based on the previous skin detection results is used to produce five regions. We firstly utilize morphological close operation to connect separate regions of previous skin detection results. Then surrounding rectangle is located from skin pixel boundary. After that, each region boundary is determined by self-defined proportions according to each facial skin scope. As seen in Figure 9, we illustrate the flowchart of local regions segmentation. Although the local region segmentation method is simple, it still works to improve recognition rate.

According to classification performance of each region in Table 2, we consider assigning five regions with different weights, higher performance, and larger weights. In our experiment, left and right cheeks are set as 0.3, forehead and nose are 0.1, and jaw is 0.2. Our weights are selected only by simple observation of each region classification performance, without optimization experiment. So those weights are probably not the best choice. Despite that, it still obtains an improvement compared with our preliminary results.

After local region weights assignment, we look for two levels of fusion strategies to perform facial color recognition: feature level fusion and classifier level fusion. To achieve feature level fusion, each region complexion histogram is constructed with its respective weight. Then regional histograms are concatenated to a holistic histogram to represent facial complexion. After histogram normalization, it is possible to perform model training and test with leave-one-out CV. For classifier level fusion, each region builds its complexion histogram and performs model training and test separately. Then the weights are used to weight vote of all predicted colors. The highest vote is selected as final facial colors.

The results of these fusion strategies are listed in Table 2; it clearly shows that fusion of local regions would achieve higher performance than any local regions and whole face without weighted local spatiality. Of these, feature level fusion would achieve highest performance. So this suggests that for facial color recognition problem, more than color-level issues should be explored, but the spatiality-level distribution of skin color should be studied by experts in order to obtain good recognition rate.

**Figure 10:**
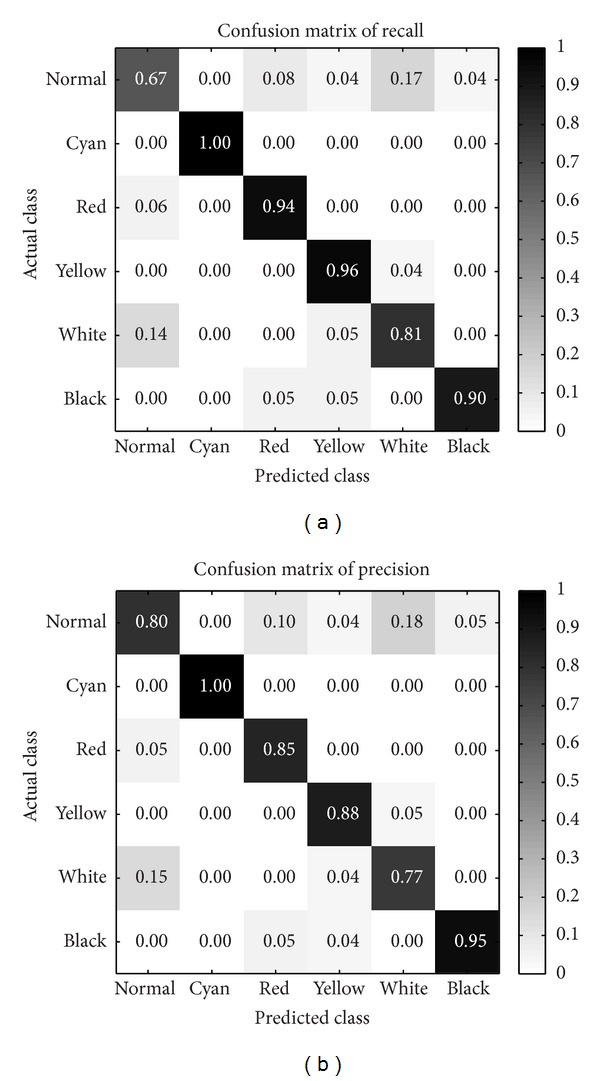
The confusion matrices produced by our improved model.

After the improvement of classification performance, we are supposed to analyze new performance on each facial color category. In Figure 10, what we observed is that the improved model could achieve higher recognition rate on red, yellow, white, and black colors. Hence, it shows why our overall classification results could reach higher performance. However, normal facial color still suffers inferior performance on recall metric. It informs us that normal color needs to be studied in-depth. In the future, how to improve the normal color recognition performance would be the focus of our researches.

### 3.5. Quantitative Results

Qualitative results show the facial color classification performance of built model. In this section, we demonstrate some quantitative analysis results for complexion degree including the color degree and the gloss degree.

Based on the features we improved in the previous section, from a total of 120 feature dimensions with five local facial regions information, we weighted each region as the previous task for color classification. Then we sum the feature values over all regions, deriving the overall facial color features which would be utilized for estimating the color and gloss degree later.

For a given set of facial color database, we can learn the ranking function from data in an unsupervised way. That is simply implemented by normalizing each chromaticity base for color degree estimation and weighted summing over all luminance values for gloss degree.

**Figure 11:**
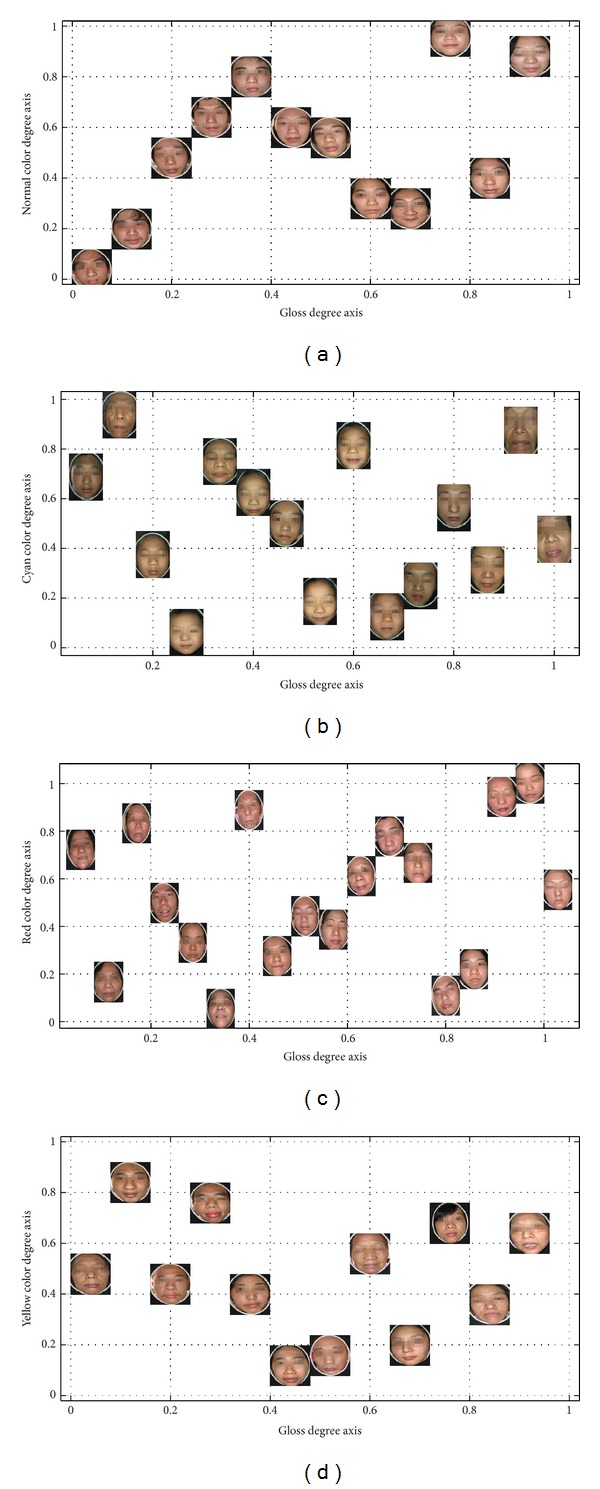
The ranking distribution according to facial color degree and gloss degree.

Once we obtain all degrees, we could also achieve the ranking distribution to show relative degrees of each patient for both facial color and gloss degrees. Therefore, in order to illustrate the learnt ranking distribution, we present a two dimensional space visualization in Figure 11. These four visualization figures are a picture subset of our facial database of four facial colors, respectively, which are projected in a two-dimensional space corresponding to the facial color and gloss dimensions.

For each complexion ranking distribution (including facial color and gloss degrees), it demonstrates a few of useful knowledge for quantitative analysis:

- the facial pictures are prone to larger facial color degree values indicate one's dramatic color reflection, hinting critical pathological changes of five internal viscera; the additional knowledge of this case would indicate easier facial color recognition, which is also validated in the previous experiments;
- pictures close to lower color degree values denote inapparent color reflection, leading to difficulty of correct recognition; in our experiments, these pictures are always incorrectly predicted by other facial colors, despite the improvement of our feature representation and model;
- in addition, based on relative degrees, pictures closed in facial color degree should have similar color category distribution in facial skin; similar pictures in color degrees also represent similar difficulty levels for facial color recognition task;
- considering the pictures distribution along gloss degree, larger degree values show the severity of specific facial color; especially for the yellow color, Yang jaundice patients would have glossy skin and lustreless skin would be reflected on the face of Yin jaundice patients. So higher gloss degree can be diagnosed as Yang jaundice, while lower gloss degree can be diagnosed as Yin jaundice; so it will be meaningful if the gloss degree distribution is well established;
- making a general analysis on two-dimensional ranking distribution, we conclude that with both higher facial color and gloss degrees, patient would be healthier for normal facial color and mild illness for morbid facial color; on the contrary, lower degrees denote serious illness and hardness to be cured.

In general, we can expect that with ranking distribution, we could firstly make some preliminary analysis for classification. This may be not only helpful to visualize feature representation but also contribute to analyze the reason of some incorrect classification results for designed feature. This is validated by our classification experiments, which incorrectly predicted that facial color is always with lower color degree values.

Moreover, for quantitative analysis, it provides both facial color and gloss degrees ranking function. In other words, we could make an in-depth analysis for each patient, examining detailed color degrees and pathological changes of five internal viscera. Sincerely, above quantitative analysis in different aspects has not been fully proved yet by TCM experts, due to unmentioned issue in the previous studies. So it is hard for us to claim that we provide authentic quantitative analysis for facial complexion. But beyond facial color recognition issue, we still make some preliminary researches on quantitative perspective. In the end, some providable quantitative conclusions are derived for deeply studying the degrees of facial color and gloss. Besides, concluded quantitative results are correlative to our previous qualitative results, interpreting why some patients are prone to be incorrectly predicted as other facial color categories.

## 4. Conclusions and Future Directions

In this paper, a novel feature representation for facial complexion (including facial color and gloss) is proposed and discussed. Based on the proposed feature, we perform extensive experiments to validate and improve its performance for facial color classification, addressed as qualitative analysis in our perspective. Both each and overall facial color classification performance are studied and discussed in our experiments. Considering the effect of parameters, the optimal luminance distribution is obtained. Furthermore, we compare the facial color information on five different local regions, respectively, and then develop improved feature representation of all hybrid regions with different weights. This result tells us that not only the chromaticity-level and luminance-level information are important, but also the spatiality-level information is useful for facial color classification. In addition, we prove that the dominant color of facial color would be extracted more reliably with our two-level clustering method. Eventually we achieve significantly improved classification performances on facial color problems.

For further researches, we also produce some quantitative analysis results for complexion degree. Although the presented ranking distribution has not been studied further on color and gloss aspects, it still shows highly correlative properties with the proposed feature for facial color classification. This may provide us with an analytical method to consider falsely predicted facial color, so it would support us to redesign a more distinctive feature representation. Besides, some preliminary quantitative conclusions are derived for further research in facial complexion diagnosis problem. However, some issues are still remaining to be addressed through current facial complexion diagnosis framework as follows.

- Although our facial image is produced under well-designed light source and camera, digital image still suffers from device-dependent color space rendering. In other words, the color information of an image is also dependent on the imaging characteristics of specific camera; it is almost impossible to be solved only by the adjustment of camera mode. Hence, more accurate color correction is necessary to be done for rendering color information into device-independent color space, as given in [24].
- In our framework, normal facial color always gets into trouble for correct recognition due to its hybrid facial colors property shown previously. Therefore, how to develop an appropriate feature representation for normal facial color would be studied in the future.
- We should also notice that the quantitative analysis presented here is preliminary in an unsupervised way. Thus, in the future work, we expect to require TCM experts to provide complexion degree ranking information of existing database, both on the color and gloss perspectives. With this prior knowledge, we could take some more outstanding supervised techniques to build complexion ranking distribution. Afterwards, more reliable quantitative analyses may be carried out on each patient.
- What has been proved in this paper is that our complexion feature representation would be more distinctive than the previous features for facial color classification problem. However, the facial gloss classification performance has not been presented here. Hence, we will study the proposed complexion feature representation for gloss classification later.
- All experiments in this paper are performed in the data set of 122 facial color images. Although our framework is effective and available in this small scale database, the performance in larger scale facial color database or in actual world is still unknown. So further experiments would be expected in future works.

## Figures and Tables

**Table 1:** Comparisons of overall performance by using all methods.

Methods	Whole face			
	Pre_macro_	Rec_macro_	*F*_macro_	*F*_micro_
Li et al.'s [1]	0.7306	0.7200	0.7252	0.7131
Liu et al.'s [12]	0.7967	0.8001	0.7984	0.7869
Wang et al.'s [13]	0.7382	0.7306	0.7344	0.7131
Our proposed method	**0.8491 **	**0.8358 **	**0.8424 **	**0.8279 **

**Table 2:** Performance comparisons using different region strategies.

Region strategies	Pre_macro_	Rec_macro_	*F*_macro_	*F*_micro_
Left cheek	0.8219	0.8274	0.8247	0.8115
Right cheek	0.8286	0.8345	0.8315	0.8197
Forehead	0.6146	0.6254	0.6200	0.5984
Nose	0.6478	0.6401	0.6439	0.6230
Jaw	0.7115	0.7327	0.7220	0.7049
Whole face (no fusion)	0.8491	0.8358	0.8424	0.8279
Classifier level fusion	0.8648	0.8688	0.8668	0.8525
Feature level fusion	**0.8758 **	**0.8798 **	**0.8778 **	**0.8689 **
